# Retinal Structure Abnormalities in Parkinson’s Disease and Atypical Parkinsonism

**DOI:** 10.3390/biom13020218

**Published:** 2023-01-23

**Authors:** Xinxin Ma, Shuhua Li, Bodi Zheng, Lei Hu, Huijing Liu, Zheng Wang, Zhaoxia Wang, Haibo Chen, Wen Su

**Affiliations:** 1 Department of Neurology, Beijing Hospital, National Center of Gerontology, Institute of Geriatric Medicine, Chinese Academy of Medical Sciences, Beijing 100730, China; 2 Department of Ophthalmology, Beijing Hospital, National Center of Gerontology, Institute of Geriatric Medicine, Chinese Academy of Medical Sciences, Beijing 100730, China; 3 Department of Neurology, Peking University First Hospital, Beijing 100034, China

**Keywords:** Parkinson’s disease, atypical parkinsonism, optical coherence tomography, multiple system atrophy, progressive supranuclear palsy

## Abstract

We investigated retinal structure changes in patients with Parkinson’s disease (PD), multiple system atrophy (MSA), progressive supranuclear palsy (PSP), and controls, and explored the value of this method in differential diagnosis. Spectral domain optical coherence tomography (SD-OCT) was used to measure peripapillary retinal nerve fiber layer (pRNFL) thickness, and macular thickness and volume. PSP patients showed higher temporal pRNFL thickness than PD and MSA patients. Peripapillary RNFL thickness could be used for discriminating PSP from MSA and PD. PD and MSA patients showed retinal thinning in the foveal center circle and nasal inner sectors compared to controls. Macular thickness and volume could be used for discriminating MSA from PD. There were negative correlations between disease duration and OCT parameters in PD, MSA, and PSP, independent of age, sex ratio, and the side of the eye. PD and atypical parkinsonism correlate with specific patterns of retina alterations. OCT could be a biomarker for differential diagnosis and progression evaluation of parkinsonian syndrome.

## 1. Introduction

Parkinsonism is characterized by the symptoms of bradykinesia or akinesia, resting tremor, rigidity, and postural instability. The most common neurodegenerative parkinsonian syndromes include Parkinson’s disease (PD), atypical parkinsonism (APS), such as multiple system atrophy (MSA), and progressive supranuclear palsy (PSP) [1,2]. PD and MSA are α-synucleinopathies, while PSP is a 4-repeat tauopathy [2,3]. The differential diagnosis between PD and atypical parkinsonism can be challenging, especially in the early stage.

Optical coherence tomography (OCT), a non-invasive and contactless method, has been increasingly used for in vivo imaging of the retina [4]. Previous studies have provided evidence supporting retina thinning in PD, MSA, and PSP [5,6,7]. A recent systematic review and meta-analysis suggested that PSP and MSA exhibited more marked peripapillary nerve fiber layer (pRNFL) thickness and central macular thickness (CMT) thinning than PD [5]. Their findings showed that OCT examination of retinal abnormalities may be an effective biomarker for PD and APS. However, the results of the various studies of OCT in PD and atypical parkinsonism have been inconclusive [8].

The aim of this study is to investigate the pRNFL thickness, and macular thickness and volume in PD, MSA, PSP, and controls, and to explore the value of OCT in the differential diagnosis of parkinsonism.

## 2. Materials and Methods

Our study recruited patients with PD, MSA, PSP, and controls from July 2018 to March 2022. All PD patients were diagnosed by experienced neurologists specializing in neurodegenerative disorders, and met the 2015 Movement Disorder Society diagnostic criteria [9]. Patients diagnosed possible and probable MSA [10] or PSP [11] according to the Movement Disorder Society (MDS) criteria were enrolled in this study. Controls underwent neurological examination and had no history of any neurodegenerative or ophthalmological disease. All patients were diagnosed by neurologists specializing in movement disorders (Haibo Chen and Wen Su). Subjects underwent a detailed clinical interview and complete neurological examination. Patient demographic and clinical information, such as age, gender, and disease duration were collected. Each patient contributed with two eyes. Patients having intraocular surgery (n = 4), age-related macular degeneration (n = 2), diabetic retinopathy (n = 3), retinal vein or artery occlusion (n = 1), epiretinal membrane (n = 1), macula hole (n = 1), high myopia, defined as corrective sphere greater than −6 diopters (n = 3), and glaucomatous optic neuropathies (n = 1), and patients incapable of undergoing OCT examination (n = 4) were excluded from the study. Therefore, 24 PD patients (48 eyes), 19 MSA patients (38 eyes), 9 PSP patients (18 eyes), and 14 controls (28 eyes) were considered in our study.

This study was approved by the local ethics committee, and written informed consent was obtained from each participant after a detailed description of the study was provided.

### 2.1. Ophthalmologic Evaluations

All participants underwent comprehensive ophthalmologic examination. Participants’ eyes with any of the following ophthalmologic pathologies were excluded: high myopia (>−6.00 diopters), high hyperopia (>−6.00 diopters), high astigmatism (≥3.00 diopters), previous intraocular surgery, or coexisting ocular disease (i.e., retinal pathology, glaucoma, shallow anterior chamber, and cataract resulting in poor-quality images). High-resolution retinal imaging was acquired using a spectral domain OCT (SD-OCT) (Spectralis; Heidelberg Engineering, Heidelberg, Germany, Software version 1.10.4.0) with the eye tracking function enabled.

Images were acquired in the seated position with the subjects facing the SD-OCT equipment. Subjects were instructed to fix their gaze on a green target during the scan. Pupils were not dilated before examination. According to OSCAR-IB criteria [12] and APOSTEL 2.0 recommendations [13], the signal strength was more than 15 dB with appropriate averaging of multiple scans (ART activated). We conducted 25 scans (ART 25) and 100 scans (ART 100) to measure peripapillary nerve fiber layer thickness and macular thickness, respectively. The numbers of scans were the same for all participants. Poor quality images were excluded. The OCT scan here was well illuminated. The laser beam was placed centrally. The line connecting the center of the optic disk and macula was correctly placed. The ring scan was correctly centered. In some patients, involuntary eye movements made the test difficult to obtain. In such cases, repeated scans were performed to obtain at least three scans without eye movement artifacts. The scan with the best resolution was used for analysis. All OCT scans were rated by one well-trained rater in our study. We used the same device in the study. All ophthalmologic examinations were completed on the same day.

### 2.2. Peripapillary Retinal Nerve Fiber Layer (pRNFL) Analysis

Peripapillary retinal nerve fiber layer (pRNFL) thickness was evaluated with three circular scans, with diameters of approximately 3.5 mm, 4.1 mm, and 4.7 mm, which were manually positioned at the center of the optic disk. RNFL boundaries were automatically delineated under the circumpapillary circle, and the pRNFL was automatically segmented. The pRNFL Spectralis protocol generated a map showing the average thickness and maps with six sector thicknesses (superonasal, nasal, inferonasal, inferotemporal, temporal, superotemporal).

### 2.3. Macular Thickness and Volumetry Analysis

Average retinal thickness and volumetry were measured in the nine macular Early Treatment Diabetic Retinopathy Study (ETDRS) areas, including a central 1 mm disk and inner and outer rings of 3 and 6 mm, respectively. A map with nine sectors was generated, including the foveal central circle, the inner ring (superior inner, temporal inner, inferior inner, and nasal inner), and the outer ring (superior outer, temporal outer, inferior outer, and nasal outer). The results were automatically calculated. The average macular thickness values at each specific quadrant were expressed in micrometers (μm), and the volumetry was expressed in cubic millimeters (mm^3^).

### 2.4. Statistical Analysis

The Statistical Package for the Social Sciences (SPSS) software (version 22.0) and GraphPad Prism software (version 7.0a) were used to was used to analyze clinical and demographic variables. Analysis of variance (ANOVA) followed by post hoc tests and chi-squared tests were conducted to examine the clinical differences between continuous and categorical variables, respectively, between four groups. We used a generalized estimating equation (GEE) model with an exchangeable structure based on a linear model, controlling for within-participant inter-eye correlations and the effect of age and sex on pRNFL, macular thickness, and volume. In order to compare the discriminatory power of the different applied imaging methods to detect different parkinsonism patients, we calculated receiver operating characteristic (ROC) curves and compared the area under the curve (AUC). In order to reduce the impact of confounding factors, we chose age, sex, and disease duration as the covariates for binary logistic regression, and then conducted the ROC analysis. Multivariate linear regression analysis was carried out between OCT parameters and disease duration in the PD, MSA, and PSP groups, respectively, controlling for age, sex ratio, and the side of the eye. In addition, one sample t-test was used to calculate the 95% confidence interval (CI) of each OCT parameter in controls. Then, we analyzed the proportion of patients in three different levels: below 95% CI, within 95% CI, and above 95% CI of controls in the three patient groups. Statistical significance was set at *p* < 0.05.

## 3. Results

### 3.1. Demographic Characteristics of All Participants

Demographic characteristics of all participants are detailed in Table 1. There were no significant differences in age (F = 0.176, *p* = 0.912) and sex ratio (χ^2^ = 6.505, *p* = 0.089) between PD, MSA, PSP, and controls. PD (range: 4 months–10 years), MSA (range: 6 months–7 years), and PSP (range: 1.5–8 years) patients differed in the mean disease duration (F = 4.314, *p* = 0.019). Post hoc tests revealed that PD showed longer disease duration than the MSA group (2.0, 95%CI: 0.55–3.39, *p* = 0.007).

### 3.2. Peripapillary Retinal Nerve Fiber Layer Thickness (the 3.5 mm Circle Surrounding the Optic Disk)

**Figure 1:**
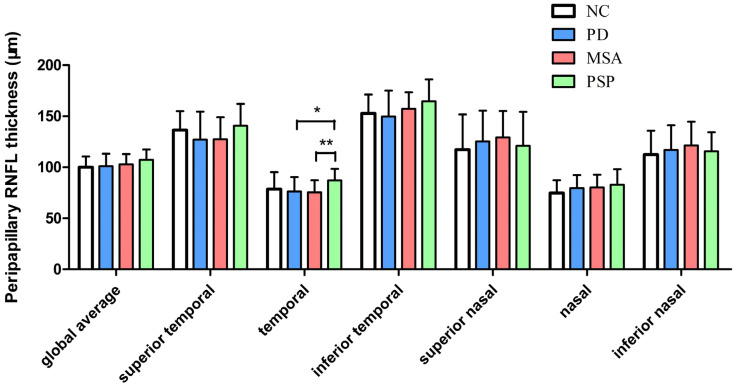
Peripapillary retinal nerve fiber layer thickness in patients with PD, MSA, PSP, and controls. * *p* ≤ 0.05, ** *p* ≤ 0.01. The error bar describes standard deviations (SD). Abbreviations: PD, Parkinson’s disease; MSA, multiple system atrophy; PSP, progressive supranuclear palsy.

Table 2 and Figure 1 show the pRNFL thickness at the 3.5 mm circle surrounding the optic disk in different groups. There was a significant difference in the temporal pRNFL thickness between the four groups (*p* = 0.036). Post hoc tests revealed that PSP patients exhibited higher pRNFL thickness than PD (10.8 μm, 95%CI: 2.29–19.36, *p* = 0.013) and MSA patients (11.7 μm, 95%CI: 3.29–20.08, *p* = 0.006) in the temporal sector.

ROC analysis for discriminating MSA from PSP found that the area under the curve (AUC) was 0.825 (95%CI 0.713–0.936, *p* < 0.001, sensitivity 83.3%, specificity 81.5%) for pRNFL thickness in the temporal sector, after controlling for age, sex ratio, and disease duration. The AUC for discriminating PD from PSP was 0.892 (95%CI 0.808–0.977, *p* < 0.001, sensitivity 77.8%, specificity 89.6%) for pRNFL thickness in the temporal sector, after controlling for age, sex ratio, and disease duration.

**Figure 2:**
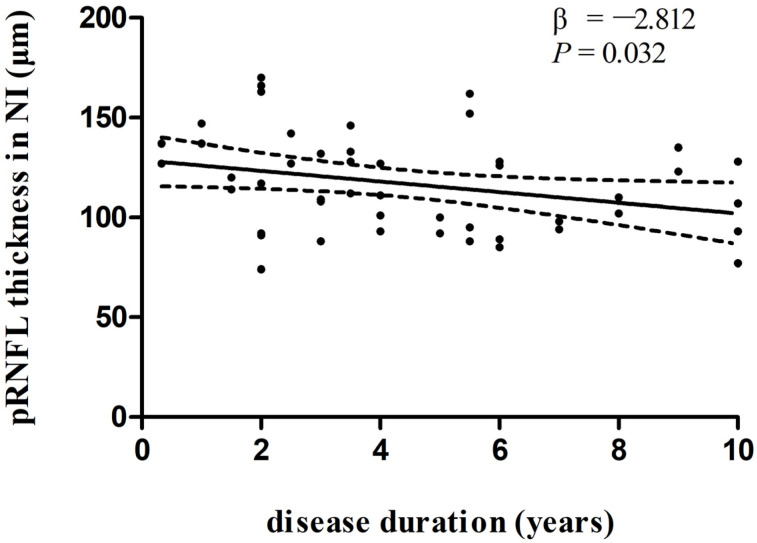
Relationships between disease duration and pRNFL thickness in the inferonasal quadrant in PD group. Trend lines describe multivariate linear regression with 95% confidence intervals (dashed line). Abbreviations: pRNFL, peripapillary retinal nerve fiber layer; NI, inferonasal sector.

**Figure 3:**
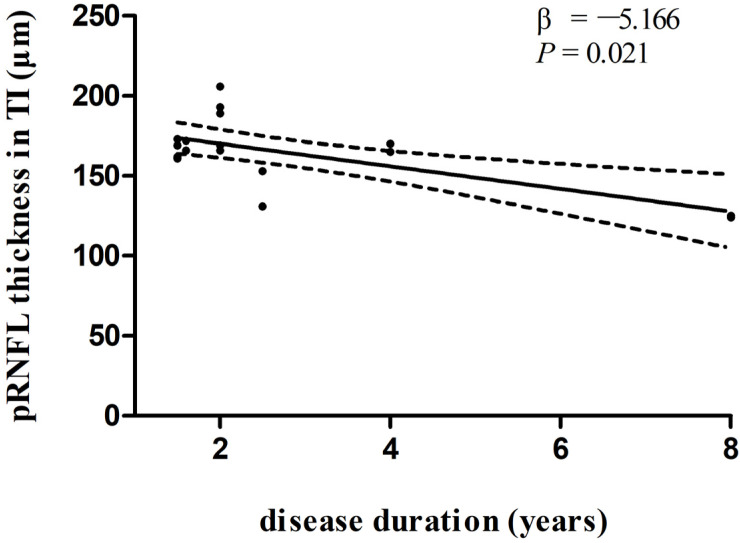
Relationships between disease duration and pRNFL thickness in the inferotemporal sector in PSP group. Trend lines describe multivariate linear regression with 95% confidence intervals (dashed line). Abbreviations: pRNFL, peripapillary retinal nerve fiber layer; TI, inferotemporal sector.

**Figure 4:**
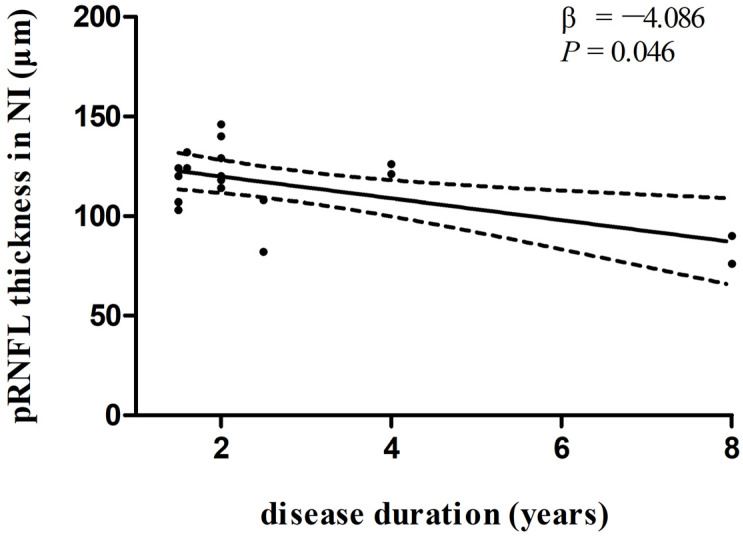
Relationships between disease duration and pRNFL thickness in the inferonasal sector in PSP group. Trend lines describe multivariate linear regression with 95% confidence intervals (dashed line). Abbreviations: pRNFL, peripapillary retinal nerve fiber layer; NI, inferonasal sector.

In multivariate linear regression analysis, there was a significant negative correlation between disease duration and pRNFL thickness in the inferonasal quadrant (Figure 2) in the PD group, independent of age, sex ratio, and the side of the eye. There were also negative associations between disease duration and pRNFL thickness in the inferotemporal (Figure 3) and inferonasal (Figure 4) sectors in the PSP group, after controlling for age, sex ratio, and the side of the eye.

**Figure 5:**
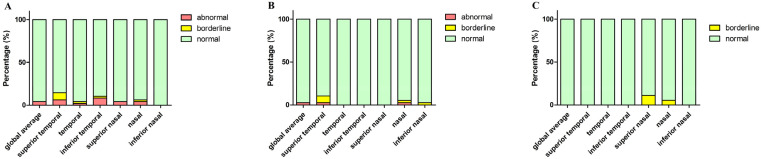
The percentage of three levels (based on Spectralis normative data) of pRNFL thickness (diameter of 3.5 mm) in PD, MSA, and PSP groups. (**A**) Parkinson’s disease; (**B**) MSA, multiple system atrophy; (**C**) PSP, progressive supranuclear palsy. Abbreviations: pRNFL, peripapillary retinal nerve fiber layer.

**Figure 6:**
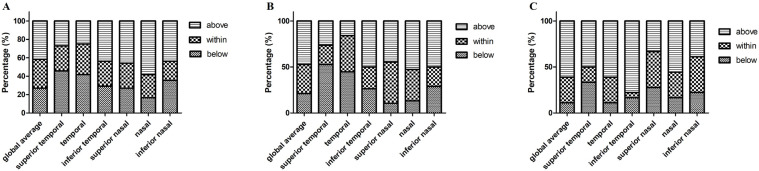
The percentage of pRNFL thickness (diameter of 3.5 mm) below, within, and above 95% confidence interval (according to the controls) in PD, MSA, and PSP groups. (**A**) Parkinson’s disease; (**B**) MSA, multiple system atrophy; (**C**) PSP, progressive supranuclear palsy. Abbreviations: pRNFL, peripapillary retinal nerve fiber layer.

According to Spectralis normative data, we divided the parameters into three levels: normal limit, borderline, and abnormal limit. Figure 5 shows the proportion of the three OCT parameters levels in the PD, MSA, and PSP groups, respectively. Macular thickness and volume in PD, MSA, and PSP patients were all within the normal range (200–250 μm). We also revealed the proportion of patients in three different levels: below, within, and above 95% CI of controls’ pRNFL thickness in the three patient groups (Figure 6).

### 3.3. Peripapillary Retinal Nerve Fiber Layer Thickness (the 4.1 mm Circle Surrounding the Optic Disk)

There was a significant difference in the global average (*p* = 0.041), inferotemporal (*p* = 0.036), and nasal (*p* = 0.022) pRNFL thickness between the four groups (*p* = 0.036). Post hoc tests revealed that PSP patients exhibited higher global average pRNFL thickness than PD (6.4 μm, 95%CI: 0.79–11.92, *p* = 0.025), MSA (5.8 μm, 95%CI: 0.25–11.32, *p* = 0.041), and controls (8.0 μm, 95%CI: 2.08–13.94, *p* = 0.008). PSP patients also showed higher pRNFL thickness than PD (15.0 μm, 95%CI: 3.20–26.87, *p* = 0.013) and controls (14.8 μm, 95%CI: 4.03–25.58, *p* = 0.007) in the inferotemporal sector. Moreover, PSP patients showed higher pRNFL thickness than MSA (6.8 μm, 95%CI: 0.23–13.28, *p* = 0.042) and controls (10.5 μm, 95%CI: 3.82–17.21, *p* = 0.002) in the nasal sector. (Table 3).

### 3.4. Peripapillary Retinal Nerve Fiber Layer Thickness (the 4.7 mm Circle Surrounding the Optic Disk)

There was a significant difference in the temporal pRNFL thickness between the four groups (*p* = 0.049). Post hoc tests revealed that PSP patients exhibited higher pRNFL thickness than PD (7.2 μm, 95%CI: 0.53–13.76, *p* = 0.034) and MSA (8.8 μm, 95%CI: 2.46–15.19, *p* = 0.007) in the temporal sector. (Table 4).

### 3.5. Macular Thickness and Volumetry Analysis

Due to poor coordination, one normal control only contributed with one eye. In terms of macular thickness (Table 5), there were significant differences in the foveal center circle (*p* = 0.002) and nasal inner sector (*p* = 0.026) RNFL thickness between the four groups. Post hoc tests revealed that MSA patients showed shorter RNFL thickness in the foveal center circle sector than controls (−28.4 μm, 95%CI: (−12.68)–(−44.03), *p* < 0.001) and PD patients (−15.0 μm, 95%CI: (−2.49)–(−27.48), *p* = 0.019). PD patients showed retinal thinning in the macular nasal inner sectors compared with controls (−11.6 μm, 95%CI: (−0.46)–(−22.68), *p* = 0.041). MSA patients exhibited shorter macular thickness in the nasal inner sectors than controls (−16.4 μm, 95%CI: (−5.64)–(−27.21), *p* = 0.003).

With respect to macular volumetry (Table 6), there were significant differences in macular volume in the foveal center circle (*p* = 0.002) and nasal inner sector (*p* = 0.023) between the four groups. Post hoc tests revealed that MSA patients showed decreased macular volume in the foveal center circle sector than controls (−0.02 mm^3^, 95%CI: [−0.010]–[−0.034], *p* < 0.001) and PD patients (−0.01 mm^3^, 95%CI: (−0.002)–(−0.022), *p* = 0.017). PD patients showed macular volume reduction in the nasal inner sector compared with controls (−0.02 mm^3^, 95%CI: (−0.001)–(−0.036), *p* = 0.039). MSA patients exhibited shorter macular thickness in the nasal inner sector than controls (−0.03 mm^3^, 95%CI: (−0.009)–(−0.042), *p* = 0.003).

ROC analysis for discriminating MSA from PD found that the AUC was 0.799 (95%CI 0.707–0.891, *p* < 0.001, sensitivity 85.4%, specificity 63.2%) for macular thickness, and 0.797 (95%CI 0.703–0.892, *p* < 0.001, sensitivity 68.1%, specificity 81.6%) for macular volume in the foveal center circle, after controlling for age, sex ratio, and disease duration.

Macular thickness in the inferior outer sector negatively correlated with disease duration in the MSA group, after controlling for age, sex ratio, and the side of the eye (β = −3.860, *p* = 0.029). There was no significant difference between macular thickness and disease duration in the PD and PSP groups, after controlling for age, sex ratio, and the side of the eye. After controlling for age, sex ratio, and the side of the eye, macular volume in the temporal outer sector negatively correlated with disease duration in the MSA group (β= −0.024, *p* = 0.029). There were no significant differences between macular volume and disease duration in the PD and PSP groups.

## 4. Discussion

Our study provided evidence for the involvement of the retina in parkinsonian disorders. Peripapillary retina thickness was significantly thinner in PD and MSA than in PSP. Macular thickness and volume were markedly thinner in MSA than those in PD. After adjusting for multiple variables, OCT parameters correlated with disease duration in patients with PD, MSA, and PSP.

The reduction in superior outer macular thickness has been demonstrated in PD patients compared with controls [14]. A meta-analysis included 36 OCT studies and showed a significant thinning of peripapillary RNFL in PD [6]. More recently, Chang et al. revealed that PD patients showed decreased pRNFL thickness in the inferior and temporal quadrants than normal controls, especially in the PD patients with cognitive impairment [15]. Some studies also demonstrated the association between the OCT parameters and disease progression in PD [16], whereas others did not yield positive results [14]. Our findings suggested that macular abnormalities were involved in PD patients. OCT could be a useful biomarker of PD, while patients with PD did not show marked reduction in pRNFL thickness compared to controls. We speculated that the reasons might be the shorter disease duration in our study and different ethnic background. The correlation between disease duration and pRNFL thickness in the inferonasal quadrant in PD was also observed in our study. This finding supported the value of OCT as a possible biomarker in monitoring PD progression.

In addition, Mendoza-Santiesteban suggested that MSA patients showed significant pRNFL thinning in all quadrants, except in the temporal quadrant, compared with normal controls [7]. The peripheral paramacular retinal thickness was also thinner in MSA patients than controls [8,17]. Another study showed that, compared with controls, MSA patients exhibited significantly reduced pRNFL thickness in the inferior and inferotemporal sectors and decreased macular thickness in the superior outer sector [18]. There were significant negative correlations between clinical features and macular thickness, including the center circle, the inferior and nasal outer sectors, and the total macular sectors [18]. Compared with controls, MSA patients showed more decreased pRNFL thickness in the superior, inferior, and nasal sectors, and central macular thickness (CMT) in the outer sectors [5]. Fisher et al. also revealed that MSA patients showed a significant pRNFL thinning in the nasal sector compared to controls, while there was no marked difference in foveal thickness between MSA and normal controls [19]. Furthermore, a longitudinal study also revealed that pRNFL tended to decrease over time in patients with MSA [20]. However, some other studies suggested that there was no significant association between pRNFL thickness and disease severity in MSA [19,21]. The present findings showed macular thickness and volume reduction in the foveal center circle and nasal inner sector in MSA patients compared to controls. Macular thickness and volume also correlated with disease duration after adjusting for confounding factors. Our study supported the suggestion that structural alterations in the retina may reflect the pattern and degree of neurodegeneration in patients with MSA. The changes in macular thickness are more sensitive than those of peripapillary RNFL in MSA.

In terms of PSP, reduction in the macular thickness in PSP has been found by an SD-OCT study, both in the peripheral and the central part of the retina, compared with controls [8]. The RNFL thickness did not correlate with the clinical parameters [8]. Stemplewitz et al. also demonstrated that PSP patients showed pRNFL thinning in the inferior nasal and inferior temporal quadrants, and decreased macular volume and thickness compared to controls [22]. Structural retinal alterations were not associated with disease duration or severity in their study [22]. Average pRNFL thickness and average central macular thickness (CMT) were thinner in PSP than controls [5]. More recently, reduction in global pRNFL thickness has been demonstrated in PSP patients. There was a correlation between pRNFL thickness and Hoehn and Yahr stages [23]. Our study showed that PSP patients might exhibit compensatory thickening of pRNFL. The mean disease duration was around two years. Therefore, we speculated that one of the reasons might be related to the relatively mild retinal degeneration in PSP.

With respect to the value of OCT in differential diagnosis of parkinsonian syndrome, the results are still controversial and limited. Albrecht et al. indicated that the mean pRNFL did not differ significantly between PD, MSA, PSP, CBS, and normal controls [8]. Subsequently, Mendoza-Santiesteban suggested M-ganglion cells were more affected in MSA, while P-ganglion cells were severely affected in PD [7,20]. More recently, a systematic review and meta-analysis suggested that average pRNFL thickness and average central macular thickness (CMT) were thinner in PSP and MSA than controls [5]. In addition, PSP and MSA revealed more marked pRNFL and CMT thinning than PD [5]. In our study, there were significant differences in pRNFL thickness and macular thickness and volume between PD, MSA, and PSP. The ROC analysis also revealed a diagnostic accuracy for pRNFL thickness in the temporal sector in discriminating between PSP and PD, and between PSP and MSA, as well as center circle macular thickness and volume in discriminating between MSA and PD.

We provide evidence for the effect of retinal imaging on the differential diagnosis and progression monitoring of parkinsonian syndromes. Several reasons may account for the different patterns of retinal alterations in PD and atypical parkinsonism (APS). First, previous observations supported the differences in preferential damage of P-cells and M-cells between PD and MSA [7,20]. P-cells predominate in the macular region projecting to the temporal area of the optic nerve. M-cells, which are mainly located in the peripheral retina, are more affected than P-cells in MSA. Hence, MSA patients revealed marked thinning in all pRNFL quadrants, except in the temporal pRNFL sector [7,20]. Second, the pathological mechanisms of PD and APS are different. MSA correlated with the deposition of misfolded α-synuclein predominantly in oligodendroglia, while PD related to α-synuclein accumulation mainly in neurons [24]. Retinal damage has been confirmed in previous pathological studies in PD and MSA [25]. With regard to PSP, accumulation of tau protein in retinal layers has been demonstrated, with possible evolution into retinal thinning [26,27]. The deposition of α-synuclein and tau protein may be different in the retina. Therefore, PD and atypical parkinsonism correlate with distinct alterations in retinal structure. Furthermore, we found that PSP showed increased pRNFL thickness in the temporal sector. Combined with previous findings [22,23], we speculate that the maculopapillar bundle fibers are relatively spared in PSP. Finally, we surmised that the patterns of retinal damage may also differ in different stages of the disease.

Several limitations should be discussed before considering the results of this study. (1) The number of participants in our study is limited, therefore, replication studies with larger sample sizes and longitudinal studies are warranted. (2) The correlations between OCT parameters and clinical features have not been analyzed, which could be conducted in the future. (3) Macular ganglion cell–inner plexiform layer complex (GCIPL) thickness reduction has been demonstrated in PD. There was also an association between GCIPL thinning and increased risk of cognitive decline in PD [28,29]. In addition, parafoveal thinning of GCIPL was observed in Lewy body disease, correlating with visual dysfunction [30]. However, we did not calculate GCIPL and other layers of the retina. That is one of the limitations in our study. We hope that further studies could be conducted to validate these findings. (4) With the current evidence, the utility of OCT in parkinsonian syndromes is only in a research context and not at the bedside. (5) Future integrated studies combining with OCT angiography (OCTA) and histopathological analysis are needed to provide new insights into the mechanisms involved in different parkinsonian disorders.

## 5. Conclusions

In summary, we found macular thinning in PD and MSA, while peripapillary RNFL thickened compensatorily in PSP. PD and atypical parkinsonism showed specific patterns of retina alterations. Our study suggested a potential role for the use of retinal imaging with OCT as a biomarker for differential diagnosis and progression evaluation in parkinsonian syndrome. Further histopathological studies are needed to validate these findings.

## Figures and Tables

**Table 1:** Demographic features of PD, MSA, PSP, and controls.

	Controls (n = 28)	PD (n = 48)	MSA (n = 38)	PSP (n = 18)	F/χ^2^	*p*
Age	67 ± 8	65 ± 10	65 ± 7	66 ± 9	0.176	0.912
Sex, M/F	14/14	34/14	20/18	4/14	6.505	0.089
Duration	NA	4.4 ± 2.8	2.5 ± 1.5	2.8 ± 2.0	4.314	0.019 ^a^
Onset, L/R/B	NA	26/12/10	8/10/20	6/8/4	7.667	0.105

Abbreviations: PD, Parkinson’s disease; MSA, multiple system atrophy; PSP, progressive supranuclear palsy; M/F, male/female; Onset, motor symptom onset; L/R/B: left, right, bilateral. A comparison between PD, MSA, and PSP. ^a^ Analysis of variance (ANOVA) between four groups.

**Table 2:** Comparisons of peripapillary retinal nerve fiber layer thickness (μm) between different groups (3.5 mm diameter).

	Controls (n = 28)	PD (n = 48)	MSA (n = 38)	PSP (n = 18)	Wald χ^2^	*p*
Global average	100.1 ± 10.6	101.0 ± 12.3	102.8 ± 10.1	107.2 ± 10.3	3.872	0.276
Superotemporal	136.4 ± 18.5	127.1 ± 27.3	127.4 ± 21.7	140.8 ± 21.2	4.959	0.175
Temporal	78.6 ± 16.6	76.2 ± 14.1	75.4 ± 11.9	87.1 ± 11.4	8.514	0.036 ^a^
Inferotemporal	152.7 ± 18.6	149.7 ± 25.4	157.2 ± 16.2	164.6 ± 21.5	3.949	0.267
Superonasal	117.2 ± 34.6	125.3 ± 30.1	129.2 ± 26.0	121.1 ± 33.1	1.687	0.640
Nasal	74.8 ± 12.4	79.6 ± 12.8	80.2 ± 12.5	82.9 ± 15.3	4.113	0.249
Inferonasal	112.4 ± 23.4	116.9 ± 24.4	121.5 ± 23.1	115.6 ± 18.7	1.503	0.682

Abbreviations: PD, Parkinson’s disease; MSA, multiple system atrophy; PSP, progressive supranuclear palsy. ^a^ Analysis of variance (ANOVA) between four groups.

**Table 3:** Comparisons of peripapillary retinal nerve fiber layer thickness (μm) between different groups (4.1 mm diameter).

	Controls (n = 28)	PD (n = 48)	MSA (n = 38)	PSP (n = 18)	Wald χ^2^	*p*
Global	85.8 ± 8.9	87.5 ± 10.0	88.0 ± 8.6	93.8 ± 7.0	8.247	0.041 ^a^
Superotemporal	123.4 ± 13.2	115.7 ± 24.8	117.4 ± 20.3	126.4 ± 19.6	3.632	0.304
Temporal	69.8 ± 14.3	68.2 ± 12.4	67.4 ± 11.0	77.2 ± 10.3	7.273	0.064
Inferotemporal	134.8 ± 15.8	134.5 ± 21.3	138.7 ± 16.6	149.6 ± 15.1	8.572	0.036 ^a^
Superonasal	94.0 ± 30.1	104.1 ± 26.2	106.9 ± 24.8	98.4 ± 22.1	2.661	0.447
Nasal	62.8 ± 10.5	67.6 ± 9.0	66.6 ± 10.4	73.3 ± 9.1	9.617	0.022 ^a^
Inferonasal	90.9 ± 21.0	94.4 ± 20.1	95.6 ± 19.5	95.4 ± 12.0	0.605	0.895

Abbreviations: PD, Parkinson’s disease; MSA, multiple system atrophy; PSP, progressive supranuclear palsy. ^a^ Analysis of variance (ANOVA) between four groups.

**Table 4:** Comparisons of peripapillary retinal nerve fiber layer thickness (μm) between different groups (4.7 mm diameter).

	Controls (n = 28)	PD (n = 48)	MSA (n = 38)	PSP (n = 18)	Wald χ^2^	*p*
Global	75.6 ± 8.4	76.8 ± 8.4	78.0 ± 7.3	80.6 ± 8.0	2.875	0.411
Superotemporal	111.1 ± 11.5	106.8 ± 21.3	108.0 ± 17.6	113.8 ± 17.4	1.683	0.641
Temporal	64.6 ± 15.5	62.0 ± 11.4	60.3 ± 9.2	69.2 ± 8.7	7.871	0.049 ^a^
Inferotemporal	122.1 ± 14.5	121.0 ± 18.1	128.2 ± 14.3	130.7 ± 18.2	4.420	0.220
Superonasal	78.5 ± 24.9	86.3 ± 20.6	91.2 ± 22.0	81.4 ± 22.6	3.408	0.333
Nasal	54.0 ± 8.9	58.1 ± 7.9	58.1 ± 9.9	60.4 ± 11.1	4.802	0.187
Inferonasal	75.8 ± 18.0	77.8 ± 19.1	80.4 ± 15.1	77.1 ± 10.6	0.834	0.841

Abbreviations: PD, Parkinson’s disease; MSA, multiple system atrophy; PSP, progressive supranuclear palsy. ^a^ Analysis of variance (ANOVA) between four groups.

**Table 5:** Macular thickness (μm) of patients with PD, MSA, PSP, and controls.

	Controls (n = 27)	PD (n = 48)	MSA (n = 38)	PSP (n = 18)	Wald χ^2^	*p*
Foveal center circle	280.4 ± 44.0	267.2 ± 31.8	252.2 ± 17.2	262.5 ± 26.9	14.679	0.002 ^a^
Superior inner	341.8 ± 21.9	334.8 ± 20.1	327.8 ± 18.1	332.8 ± 19.0	5.838	0.120
Temporal inner	333.8 ± 26.4	323.9 ± 19.0	319.5 ± 17.1	321.1 ± 20.7	5.154	0.161
Inferior inner	341.5 ± 22.0	330.5 ± 21.6	329.8 ± 22.8	326.8 ± 19.6	5.503	0.138
Nasal inner	346.7 ± 18.6	335.1 ± 20.4	330.3 ± 19.6	335.9 ± 19.1	9.282	0.026 ^a^
Superior outer	297.2 ± 15.9	292.2 ± 17.0	290.8 ± 15.2	290.6 ± 13.4	2.068	0.558
Temporal outer	282.8 ± 17.4	277.2 ± 14.8	276.4 ± 13.3	272.8 ± 16.4	2.647	0.449
Inferior outer	282.3 ± 14.8	277.8 ± 15.4	279.0 ± 14.3	276.3 ± 19.6	1.243	0.743
Nasal outer	314.1 ± 14.2	309.6 ± 17.4	306.2 ± 15.0	307.6 ± 20.8	2.952	0.459

Abbreviations: PD, Parkinson’s disease; MSA, multiple system atrophy; PSP, progressive supranuclear palsy. ^a^ Analysis of variance (ANOVA) between four groups.

**Table 6:** Macular volume (mm^3^) of PD, MSA, and PSP patients and controls.

	Controls (n = 27)	PD (n = 48)	MSA (n = 38)	PSP (n = 18)	Wald χ^2^	*p*
Foveal center circle	0.22 ± 0.03	0.21 ± 0.02	0.20 ± 0.02	0.21 ± 0.02	14.363	0.002 ^a^
Superior inner	0.54 ± 0.03	0.52 ± 0.03	0.51 ± 0.03	0.52 ± 0.03	6.909	0.075
Temporal inner	0.52 ± 0.04	0.51 ± 0.03	0.50 ± 0.03	0.50 ± 0.03	5.675	0.129
Inferior inner	0.54 ± 0.04	0.52 ± 0.03	0.52 ± 0.03	0.51 ± 0.03	5.544	0.136
Nasal inner	0.54 ± 0.03	0.52 ± 0.03	0.52 ± 0.03	0.53 ± 0.03	9.539	0.023 ^a^
Superior outer	1.58 ± 0.08	1.55 ± 0.09	1.53 ± 0.08	1.54 ± 0.07	3.412	0.332
Temporal outer	1.50 ± 0.09	1.47 ± 0.08	1.46 ± 0.08	1.45 ± 0.08	3.267	0.352
Inferior outer	1.50 ± 0.08	1.47 ± 0.08	1.48 ± 0.08	1.46 ± 0.10	1.129	0.770
Nasal outer	1.66 ± 0.08	1.64 ± 0.09	1.63 ± 0.10	1.63 ± 0.11	1.948	0.583
Total	8.60 ± 0.44	8.42 ± 0.45	8.34 ± 0.36	8.35 ± 0.47	4.227	0.238

Abbreviations: PD, Parkinson’s disease; MSA, multiple system atrophy; PSP, progressive supranuclear palsy. ^a^ Analysis of variance (ANOVA) between four groups.

## Data Availability

The data supporting this study are available in the manuscript. Other data and images used for the assessments described in the study can be made available upon request to the corresponding author.
